# The Shab family potassium channels are highly enriched at the presynaptic terminals of human neurons

**DOI:** 10.1016/j.jbc.2025.108235

**Published:** 2025-01-27

**Authors:** Orion Benner, Charles H. Karr, Astrid Quintero-Gonzalez, Michael M. Tamkun, Soham Chanda

**Affiliations:** 1 Biochemistry & Molecular Biology, Colorado State University, Fort Collins, Colorado, USA; 2 Biomedical Sciences, Colorado State University, Fort Collins, Colorado, USA; 3 Molecular, Cellular & Integrated Neurosciences, Colorado State University, Fort Collins, Colorado, USA; 4 Cell & Molecular Biology, Colorado State University, Fort Collins, Colorado, USA

**Keywords:** potassium channel, Shab family, Kv2.1, Kv2.2, AMIGO, ER–PM junction, human neurons, presynaptic terminals

## Abstract

The Shab family voltage-gated K^+^ channels (*i.e.*, Kv2.1, Kv2.2) are widely expressed in mammalian brain and regulate neuronal action-potential firing. In addition to their canonical functions, the Kv2 proteins help establish direct attachments between plasma membrane and endoplasmic reticulum (ER), also known as ER–plasma membrane junctions. However, the biochemical properties and molecular organization of these ion channels have not yet been described in human neurons. Here, we have performed a systematic analysis of endogenous expression, post-translational modification, and subcellular distribution of the major components of Kv2 complex in neurons derived from human stem cells. We found that both Kv2.1, Kv2.2, and their auxiliary subunit AMIGO1 are significantly upregulated during early neurogenesis, localize at the cell surface, and already begin to assemble with each other. Human Kv2.1 and AMIGO1, but not Kv2.2, undergo substantial post-translational modification including phosphorylation and/or N-linked glycosylation. Acute pharmacological inhibition with Kv2 blockers also revealed their functional activation in human neurons. These proteins formed prominent clusters at cell bodies, dendritic branches, and axon initial segments. Interestingly, a large fraction of them also exhibited considerable accumulation at human presynaptic terminals, where they aggregated with the local ER network. This synaptic localization of Kv2 subunits was primarily restricted to presynaptic regions, as they demonstrated limited enrichment at postsynaptic densities. These results were highly reproducible in multiple stem cell lines used and alternative differentiation protocols tested, confirming that human presynaptic compartments can actively recruit the Shab family K^+^ ion channels.

The Shab family K^+^ channels include two major subtypes, Kv2.1 and Kv2.2. These proteins form both homomeric and heteromeric complexes (1, 2, 3, 4, 5, 6), which also recruit auxiliary subunit amphoterin-induced gene and ORF (AMIGO) 1, which is a cell-adhesion molecule comprised of leucine-rich repeats and an immunoglobulin domain (7, 8, 9). In the mammalian nervous system, Kv2 channels act as traditional delayed rectifiers (10, 11, 12, 13, 14, 15, 16, 17), essentially modulating neuronal excitabilities by contributing to the repolarization phase of action potentials during high-frequency firing (18, 19, 20, 21). Genetic mutations in these proteins have been directly linked with severe psychiatric diseases, including epilepsy, autism, cognitive defects, intellectual disability, and other neurodevelopmental disorders (22, 23, 24, 25, 26), which predominantly impair their expression levels, or the gating and conductance parameters of Kv2 channel complex.

In addition to their conducting activities, the intracellular C-terminal domain of Kv2 channels at the plasma membrane (PM) can directly interact with vesicle-associated membrane protein–associated proteins (VAPs) situated in the smooth endoplasmic reticulum (ER), which facilitates the formation of ER–PM junctions (27, 28, 29, 30, 31, 32). These ER–PM contact sites can be visualized as large Kv2 surface clusters, which play important nonconducting roles in cellular physiology. They are known to serve as local hubs for lipid exchange, membrane protein delivery, organelle biogenesis, or Ca^2+^ homeostasis (33, 34, 35, 36, 37) and are modified by signaling pathways (38, 39). The ER–PM contacts can also serve as designated sites for neuroglial interaction (40, 41, 42). Targeted disruption of Kv2–VAP binding is neuroprotective in an ischemic stroke model by reducing excitotoxic injury (43).

In rodent hippocampal or cortical pyramidal neurons, these Kv2-dependent ER–PM junctions often develop at the cell soma, where they also recruit L-type voltage-gated Ca^2+^ channels, enhancing their spatial proximity with ER-associated ryanodine receptors (RyRs). These structures drive local Ca^2+^ storage and release that promote transcriptional coupling with neuronal activities (44, 45, 46). In addition, a fraction of Kv2 clusters has been reported to extend beyond neuronal cell bodies and distribute at proximal dendrites or axon initial segments (AISs) (29, 47, 48). Nevertheless, it remains mostly unclear whether the individual elements of this Kv2 channel complex can traffic even farther, organize at more distant compartments of neurons, and interact with distal ER network.

Intriguingly, anatomical studies in rodent brain revealed a continuous and elaborate distribution of ERs that expand throughout neurons, forming discrete ER–PM junctions not only at somatodendritic areas but also at axon terminals (49, 50). In accordance, a single study has recently demonstrated that Kv2 proteins also concentrate at the presynaptic terminals of rodent primary neurons, where they do not influence the axonal action potential but are required for neurotransmitter release (51). Although these results provide a strong correlative evidence for Kv2-mediated ER–PM contacts at presynapses, it remains highly debated and requires further validations (52). Moreover, despite the extensive analyses of Kv2 channels in rodents, their molecular properties or subcellular distributions have not yet been characterized in human neurons because of the lack of a suitable model system.

In this current study, we performed a detailed and comprehensive assessment of the primary components that constitute the Kv2 complex in neurons derived from human stem cells. Our results provide critical insights into various aspects of these principal ion channels, for example, endogenous expression, surface PM trafficking, subunit interaction, voltage-dependent functional activation, and post-translational modification (PTM), as well as their subcellular distribution, and ER association. Importantly, here, we demonstrated that the human Kv2 complex can also efficiently localize to presynaptic terminals but not postsynapses, irrespective of neural differentiation protocols. Our approach offers a new avenue to further investigate the Kv2 channel biology, their conducting and nonconducting roles in neuronal physiology and pathology, particularly in a human cellular environment.

## Results

### Human neurons express several components of the Kv2 complex

**Figure 1:**
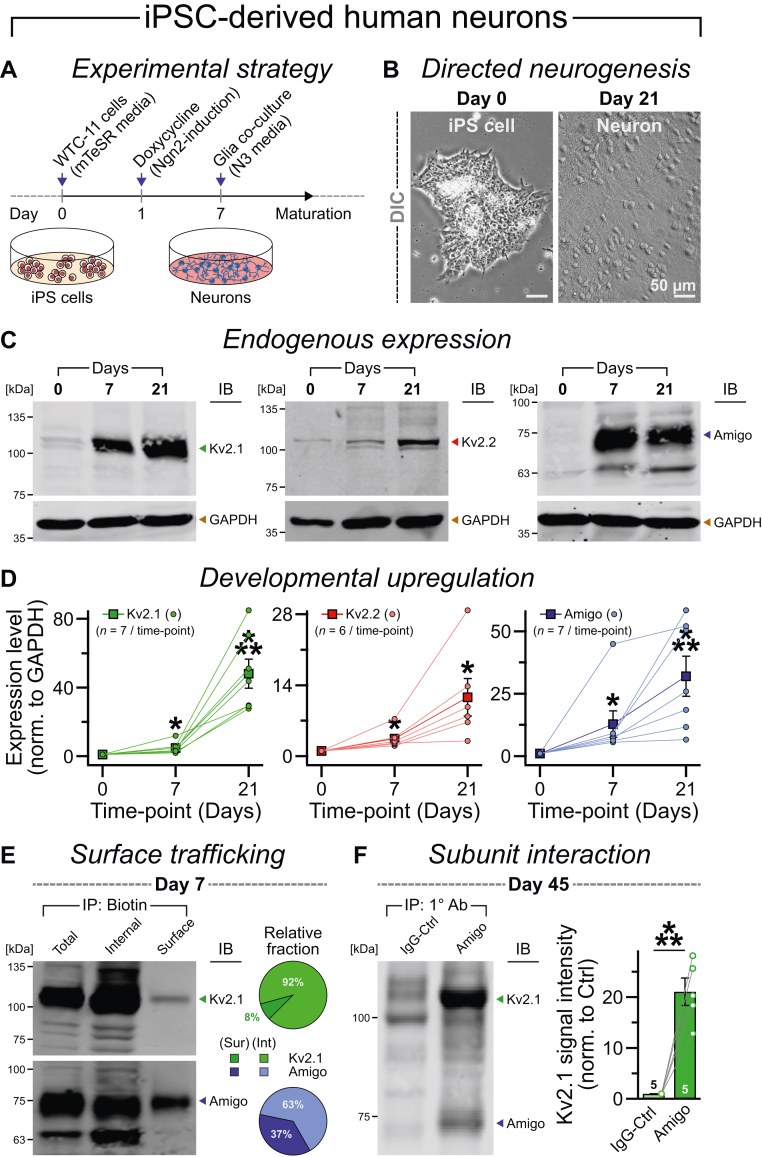
**Expression of Kv2 proteins in iPSC-derived human neurons.***A* and *B*, schematic diagram (*A*) and representative images (*B*; differential interference contrast [DIC]) of an Ngn2-inducible iPSC line (*i.e.*, WTC-11, see the *Experimental procedures* section) reprogrammed into human neurons by doxycycline treatment, cocultured with mouse glia, and analyzed at different developmental stages. *C* and *D*, example of immunoblots (IBs; *C*) and average expression levels (*D*) of endogenous Kv2.1 (*left*), Kv2.2 (*middle*), and AMIGO1 (*right*) proteins at postinduction day 0 (*i.e.*, undifferentiated stem cells), day 7, or day 21 (*i.e.*, early neurodevelopmental stages). GAPDH was used as a loading control for normalization. *E*, sample Western blots (*left*) and average percentages (*right*, pie charts; *n* = 2) of Kv2.1 (*top*) and AMIGO1 (*bottom*) subunits isolated from whole cell (total), intracellular (Int = internal), or cell surface (Sur = surface) fractions, when day 7 neurons were biotinylated under nonpermeable conditions (see the *Experimental procedures* section). *F*, example of blot (*left*) and average values (*right*) of Kv2.1 band intensity when immunoprecipitated (IP) from day 45 neuronal lysates using a control (Ctrl) immunoglobulin G (IgG) *versus* equal amount of AMIGO1 primary antibody (1° Ab). All quantifications reflect means ± SEM, with values in the *insets* indicating total number of independent biological replicates. Single data points are plotted as *color-matched, connected symbols*, with averages. Statistical significance was estimated by two-tailed, paired, Student’s *t* test, with ∗∗∗*p* < 0.005; ∗*p* < 0.05. iPSC, induced pluripotent stem cell; Ngn2, Neurogenin-2.

To monitor the endogenous expression of Kv2 ion channels during neurogenesis, we reprogrammed human-induced pluripotent stem cells (iPSCs; WTC-11 line) into predominantly glutamatergic neurons of cortical layer 2-3 identity, by forced expression of a single transcription factor Neurogenin-2 (Ngn2; (53, 54)) and then cocultured them with mouse astrocytes for further maturation (Fig. 1, *A* and *B*). We lysed the cells at day 0 (*i.e.*, stem cells stage, prior to differentiation) or postinduction days 7 and 21 (*i.e.*, after neurogenesis) and performed quantitative immunoblots with the extracted proteins (Fig. 1*C*). We found that all major components of the Kv2 complex, for example, Kv2.1 and Kv2.2, as well as auxiliary subunit AMIGO1, showed a robust increase in expression already by day 7, which continued to rise even more by day 21, as the neurons matured over time (Fig. 1, *C* and *D*).

**Figure 2:**
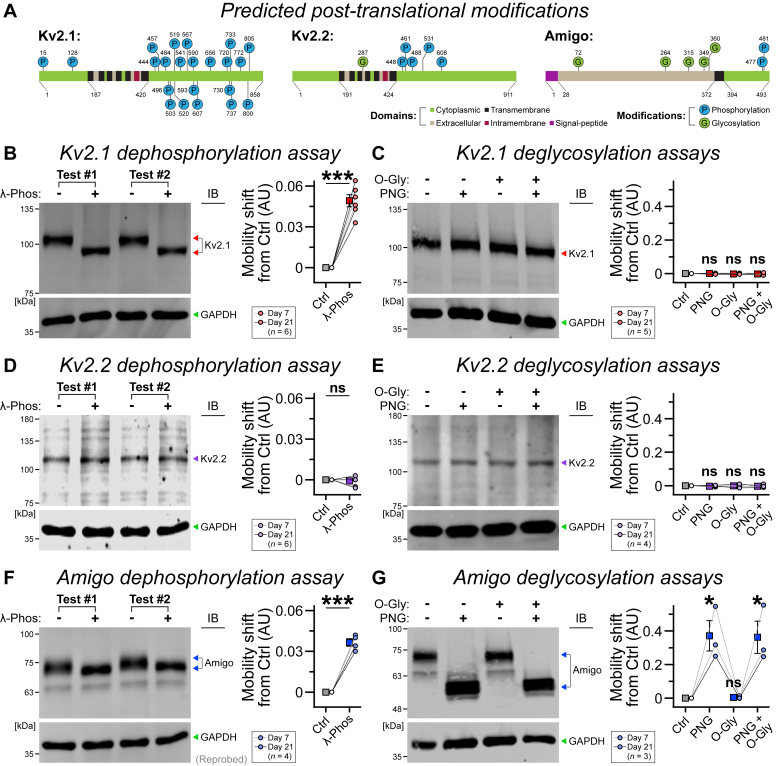
**Post-translational modifications (PTMs) of the human Kv2 complex.***A*, linearized domain maps of Kv2.1 (*left*), Kv2.2 (*middle*), and AMIGO1 (*right*) subunits, and their amino acid residues for potential phosphorylation and N-linked glycosylation, as predicted by UniProt sequence analyses. *B*, representative Western blot (*left*) and average shift in Kv2.1 band mobility (*right*), without any treatment (−) or when incubated with λ-phosphatase (+). For sample blot, two individual batches were run consecutively to ensure reproducibility; whole cell lysates from days 7 and 21 exhibited similar trends, and therefore, were averaged together. Relative distances between the control (Ctrl) *versus* treatment bands were presented with an arbitrary unit (AU). GAPDH = loading control, showed no measurable change in molecular weight because of phosphorylation. *C*, example of blot (*left*) and average shift in Kv2.1 band position (*right*) when treated with O-glycosidase (O-Gly) or PNGase F (PNG); note that glycosidase treatments did not impact Kv2.1 mass. GAPDH = loading control. *D* and *E*, same as *B* and *C*, except for Kv2.2; no obvious mobility shift was detected with phosphatase or glycosidase. *F* and *G*, same as *B* and *C*, except for AMIGO1; both λ-phosphatase and PNG but not O-glycosidase treatment demonstrated significant shift in molecular weight. Note that the same immunoblot in *D* was stripped and reprobed for *F* to highlight the contrasting PTM pattern between Kv2.2 *versus* AMIGO1, within the same experiment. All summary graphs represent means ± SEM, and the numbers of independent batches (*n*) are reported in the *insets*. All individual data points are included as *color-coded circles* adjoined by *lines* for pairwise comparison, together with average values provided as *filled squares*. Statistical powers between experimental groups were evaluated using two-tailed, paired, Student’s *t* test (∗∗∗*p* < 0.005; ∗*p* < 0.05; ns = not significant, *p* > 0.05).

To determine whether newly synthesized Kv2 subunits traffic to the cell membrane, we acutely biotinylated all surface proteins of live day 7 human neurons under nonpermeabilized conditions, affinity purified them using NeutrAvidin beads, ran Western blots, and probed them with Kv2.1 and AMIGO1 antibodies. We observed that a noticeable portion of both Kv2.1 and AMIGO1 proteins were located at the cell surface as early as day 7 after neuronal differentiation (Fig. 1*E*). The biotinylated fraction of AMIGO1 was more abundant than Kv2.1, possibly because of its much larger extracellular domain (Fig. 2*A*). To further explore the molecular affinity between these different Kv2 subunits, we performed a coimmunoprecipitation assay on day 45. We found that, compared with an immunoglobulin G control, the AMIGO1 antibody successfully isolated a large amount of Kv2.1 proteins, confirming their mutual interaction in the human Kv2 complex (Fig. 1*F*). Thus, iPSC-derived neurons offered an ideal system to analyze the molecular properties and subcellular distribution of Kv2 channels, in human cellular environment.

### Human Kv2 proteins undergo major PTM

Of note, the predicted molecular weights (MWs) of human Kv2.1, Kv2.2, and AMIGO1 are ≈96, 103, and 55 kDa, respectively. However, they generated higher MW products (Fig. 1), likely reflecting PTMs, for example, phosphorylation or glycosylation, as anticipated by sequence analysis (see UniProt; Fig. 2*A*). To test this hypothesis, we next incubated the cell lysates with λ-phosphatase, O-glycosidase, or/and PNGase F enzymes, which specifically remove phosphate groups, O-linked and N-linked oligosaccharides from proteins. We ran immunoblots with the samples and probed with Kv2.1, Kv2.2, and AMIGO1 antibodies.

We found that λ-phosphatase treatment of Kv2.1 caused a significant reduction in MW (Fig. 2*B*). However, O-glycosidase and PNGase F alone or even both enzymes combined failed to alter its band position (Fig. 2*C*). Hence, human Kv2.1 is predominantly modified by phosphorylation but not glycosylation, highly consistent with those observed in rodent models (31, 55, 56, 57, 58). Interestingly, despite a number of PTM sites predicted for human Kv2.2, neither phosphatase nor glycosidase exposure could significantly change its mobility, indicating that this protein is not effectively modified in newly born neurons (Fig. 2, *D* and *E*), in contrast with PKC-mediated phosphorylation events anticipated for Kv2.2, in human embryonic kidney cells and mouse cortical slices (59). Intriguingly, both λ-phosphatase and PNGase F, but not O-glycosidase, treatments manifested prominent shifts in AMIGO1 mobility (Fig. 2, *F* and *G*). These subunit-specific PTMs in both Kv2.1 and AMIGO1, or their absence in Kv2.2, were similarly detected on days 7 and 21 (Fig. 2, *B*–*G*). In sum, these results suggest that individual components of the Kv2 complex can undergo differential PTMs and are already mature at an early neurodevelopmental stage.

### Human Kv2 proteins can be activated in voltage-dependent manner

**Figure 3:**
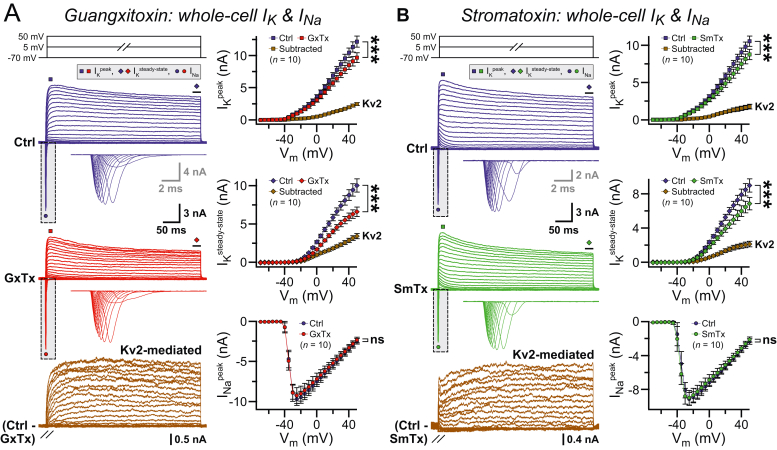
**Voltage-dependent activation of human Kv2 ion channels.***A*, example of traces (*left*) and average values (*right*) of the whole-cell I_K_ and I_Na_ recorded from day 45 to 50 human neurons, when subjected to step pulses (command voltages, *top left*), either before (control, Ctrl) or after bath application of guangxitoxin (GxTx, 100–200 nM) and their subtracted values (*i.e.*, Ctrl-GxTx) indicating Kv2-mediated sustained outward currents (*bottom left*); values were calculated for I_K_ peak (*squares*) or steady state (*diamonds*, last 25 ms), and I_Na_ (*circles*); *insets* = *dotted boxes* magnified to the *right* to visualize inward I_Na_. *B*, same as *A*, except for Kv2-mediated I_K_ isolated by acute stromatoxin (SmTx, 100–200 nM) application. All average plots (*i.e.*, *connected symbols*) reflect means ± SEM and are provided with number of neurons patched from three to four independent batches. Statistical tests for I_K_ and I_Na_ between Ctrl *versus* drug conditions (GxTx and SmTx) were conducted by two-way ANOVA with replicates (∗∗∗*p* < 0.005; ns = not significant, *p* > 0.05).

To gauge if the Kv2 proteins assemble into functional units, we next conducted electrophysiological recordings from more mature day 45 to 50 neurons and measured whole-cell K^+^ and Na^+^ currents (I_K_ and I_Na_, respectively). When subjected to depolarized membrane potential, these Ngn2-induced neurons demonstrated both outward I_K_ and inward I_Na_, as described earlier (Fig. 3*A*; (53, 60)). To estimate the relative contributions of Kv2 channels in total I_K_, we bath-applied a Kv2-selective voltage sensor modulator guangxitoxin (20, 61), while continuing to record from them. We observed that guangxitoxin application significantly decreased both the peak amplitude and the steady-state levels of whole-cell I_K_, without affecting the I_Na_ (Fig. 3*A*). Thus, our experiments revealed a prominent, outward, Kv2-mediated I_K_ component. These Kv2-mediated I_K_ components could be reproducibly detected by using a different Kv2-specific antagonist stromatoxin (Fig. 3*B*, (62)). Our results corroborate that the major Kv2 subunits successfully constitute conducting K^+^ channels in iPSC-derived human neurons.

### Kv2 accumulates at distinct subcellular regions including synapses

**Figure 4:**
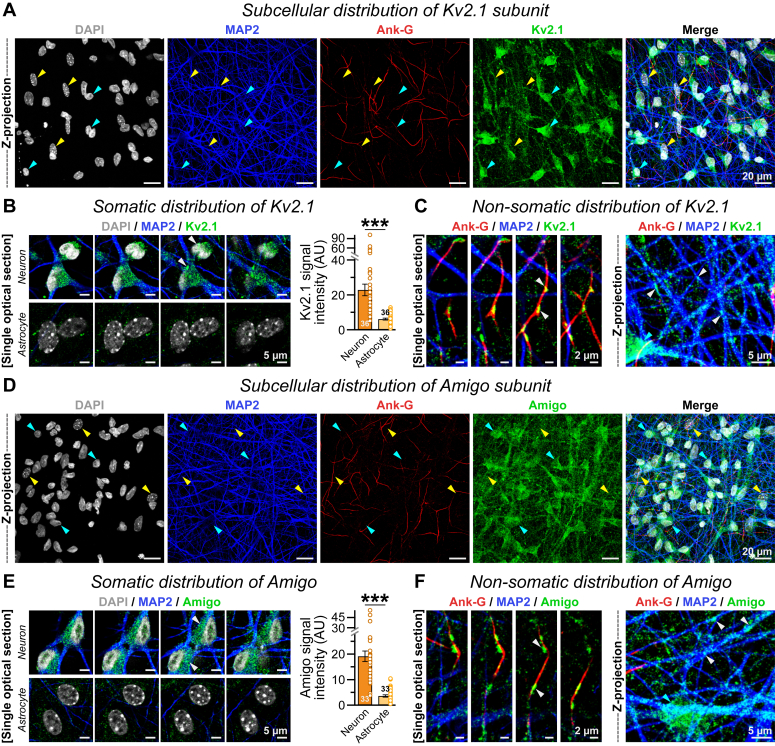
**Subcellular distribution of Kv2 complex in human neurons.***A*, day 45 human neuronal cultures were labeled with nuclear DAPI and immunostained for dendritic MAP2, Ankyrin-G (Ank-G) at AIS, the Kv2.1 subunit, and all views merged (*left to right*). *Cyan arrowheads* = MAP2-positive human neurons, *yellow arrowheads* = mouse astrocytes with heterochromatin spots in DAPI channel. *B*, example of images (*left*) and average intensities (*right*) of endogenous Kv2.1 signals from MAP2-positive neurons *versus* MAP2-negative astrocytes present in the same culture. *Arrowheads* = Kv2.1 clusters in soma. *C*, nonsomatic Kv2.1 signals (*white arrowheads*) accumulated within MAP2-negative but Ank-G-positive AIS (*left*) or along MAP2-positive dendrites (*right*), away from the clusters in neuronal cell body (*cyan arrowhead*). *D*–*F*, same as *A*–*C*, except for the Kv2 auxiliary subunit AMIGO1, which exhibited identical cellular distribution. The bar graphs represent means ± SEM, with number of images analyzed from three to four independent experimental replicates, and individual data points (*color-matched open circles*). Statistical assessments across neuron *versus* astrocyte cell types (in *B* and *E*) were performed by two-tailed, unpaired, Student’s *t* test, with ∗∗∗*p* < 0.005. AIS, axon initial segment; DAPI, 4',6-diamidino-2-phenylindole; MAP2, microtubule-associated protein 2.

We subsequently aimed to examine the subcellular localization of these Kv2 channel complexes. To this end, we immunolabeled the mature day 45 to 50 neurons with Kv2.1 antibody, along with dendritic MAP2 (microtubule-associated protein 2), Ankyrin-G for AIS, and nuclear 4',6-diamidino-2-phenylindole (DAPI). We found that human Kv2.1 largely accumulated at DAPI-positive cell bodies (Fig. 4*A*). Importantly, this somatic enrichment of Kv2.1 signals was mostly restricted to neurons, with very limited and nearly background-level expression in MAP2-negative astrocytes that were cultured together (Fig. 4, *A* and *B*). Hence, the pronounced upregulation in human Kv2 subunit expression during the *in vitro* maturation of human neurons did not arise from contaminating mouse proteins present in cocultured astrocytes (Fig. 1).

In addition, we also detected a sizable amount of nonsomatic Kv2.1 clusters on MAP2-positive proximal dendrites, some of which even colocalized with Ankyrin-G signals confirming their recruitment at the AIS (Fig. 4*C*). Nevertheless, a considerable pool of Kv2.1 proteins was targeted to more distant dendritic regions, which did not associate with either soma or AIS (Fig. 4*C*). These neuron-specific expression profiles and subcellular distribution patterns were virtually identical for the auxiliary subunit AMIGO1 (Fig. 4, *D*–*F*), implying that multiple components of the Kv2 complex are copresent at the same cellular compartments of human neurons.

**Figure 5:**
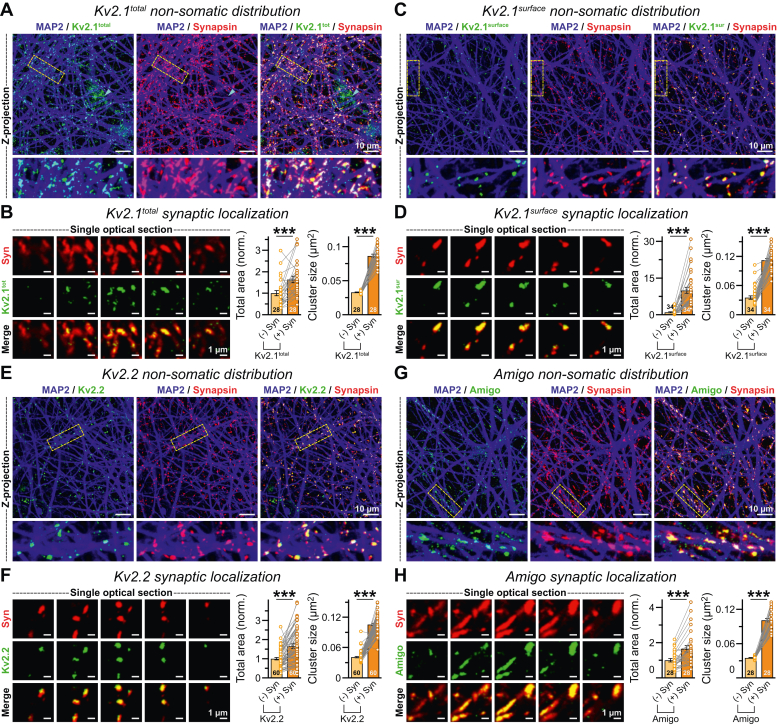
**Synaptic localization of the Kv2 proteins in human neurons.***A*, mostly nonsomatic region of a day 45 neuronal culture immunostained with MAP2, Kv2.1, and synapsin antibodies; *insets* = boxed sections magnified below for better visualization. *Arrowhead* = a single cell body. *B*, representative images (*left*) of single optical sections (0.5 μm intervals) depict synapsin and nonsomatic Kv2.1 signals; total area and average size of Kv2.1 clusters (*right*) situated outside (−) *versus* inside (+) synapsin. *C* and *D*, same as *A* and *B*, except for a different Kv2.1 antibody raised against its extracellular epitope. Note that the neurons were first immunolabeled for surface Kv2.1 population under nonpermeabilized conditions, followed by Triton X-100 permeabilization and coimmunostaining for MAP2 and synapsin (see the *Experimental procedures* section). *E* and *F*, same as *A* and *B*, except for colocalization between synapsin and nonsomatic clusters of Kv2.2 subunit. *G* and *H*, same as *A* and *B*, except for colocalization between synapsin and nonsomatic AMIGO1 subunit clusters. Summary plots are shown as means ± SEM, with total number of images (*n*) analyzed from three to four independent batches. Data points from each experiment are provided as *color-coded open circles* connected with *straight lines*. Statistical significances were calculated using two-tailed, paired, Student’s *t* test, with ∗∗∗*p* < 0.005. MAP2, microtubule-associated protein 2.

To inquire whether some of these nonsomatic Kv2 clusters could be associated with synaptic structures, we immunostained the neurons for synapsin, in combination with Kv2.1, Kv2.2, and AMIGO1 antibodies. We observed that a major fraction of endogenous Kv2.1 strongly overlapped with synapsin puncta, away from the cell body (Fig. 5*A*). Furthermore, the total area of these nonsomatic Kv2.1 signals and size of their individual clusters were significantly enhanced when recruited at synapses (Fig. 5*B*). This synaptic enrichment of Kv2.1 was also detected when nonpermeabilized neurons were labeled with a different antibody raised against its extracellular epitope, arguing against any nonspecific phenotype (Fig. 5, *C* and *D*). To further assess whether this phenomenon was limited to Kv2.1 only, we monitored the localization of other two major Kv2 subunits, Kv2.2 and AMIGO1. Once again, both Kv2.2 and AMIGO1 proteins also exhibited a very similar distribution pattern, as they effectively coaggregated with synapsin signals at distal dendritic regions (Fig. 5, *E*–*H*). To summarize, these results corroborate a preferential accumulation of the Shab family K^+^ channels at human synapses.

### Kv2 complex localizes at the presynaptic but not postsynaptic compartment

**Figure 6:**
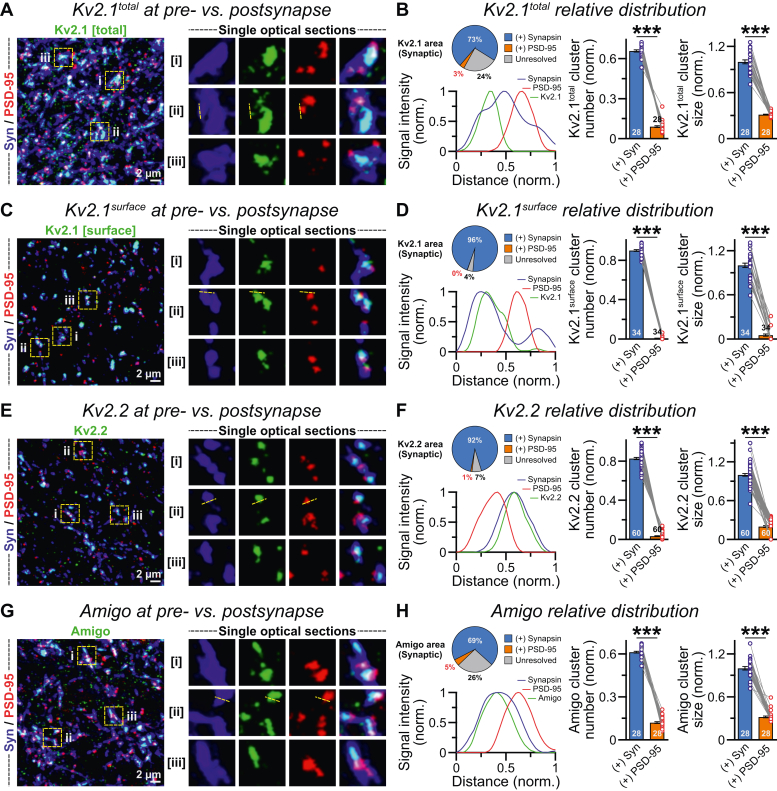
**Presynaptic but not postsynaptic recruitment of Kv2 complex.***A*, Z-projected sample image (*left*) of day 45 human neuronal cultures coimmunostained with Kv2.1 antibody (intracellular epitope), paired with both presynaptic synapsin and postsynaptic PSD-95 antibodies; the *boxed sections* (regions of interests [ROIs]: i, ii, and iii) were further enlarged (*right*) and displayed as single optical sections to illustrate relative distribution of each channel or their superimposed views at single synapse level. *B*, percentages of Kv2.1 signals (*pie charts*) colocalized with either synapsin or PSD-95 or a fraction that could not be resolved by confocal microscopy because of signal proximity; note that the Kv2.1 clusters primarily associated with synapsin, adjacent to mostly nonoverlapping PSD-95 signals (intensity profiles for optical section ii, *dotted yellow lines* in the ROI). Bar graphs reflect the average numbers and total area of Kv2.1 clusters (normalized to total signals) per field of view coinciding with either synapsin or PSD-95 puncta. *C* and *D*, same as *A* and *B*, except for Kv2.1 at cell surface recognized by its extracellular epitope. *E* and *F*, same as *A* and *B*, except when labeled with an antibody against the human Kv2.2 subunit. *G* and *H*, same as *A* and *B*, except when labeled for the AMIGO1 subunit of human Kv2 complex. Average data are presented as means ± SEM, along with the total number of field of views obtained from three to four independent experimental batches. All individual data points are provided as *colored circles* joined with *lines*, together with their corresponding averages. The statistical comparisons for Kv2 channel subunits colocalized with synapsin *versus* PSD-95 puncta were performed using two-tailed, paired, Student’s *t* test, with ∗∗∗*p* < 0.005.

Since Kv2 subunits showed strong colocalization with the presynaptic marker synapsin, we wondered if they can similarly aggregate at the postsynaptic interface. To obtain a better resolution at single synapse level, we immunostained the neurons with PSD-95 antibody that labels glutamatergic postsynaptic density. Surprisingly, we noticed that the vast majority of nonsomatic Kv2.1 channels almost exclusively clustered within subregions of synapsin signal but away from corresponding PSD-95 puncta located adjacently (Fig. 6, *A* and *B*). Moreover, the relative fraction of Kv2.1 signals and the average size of Kv2.1 clusters were considerably diminished at PSD-95 areas, compared with synapsin (Fig. 6*B*). Once again, this phenomenon was fully reproducible with a Kv2.1 extracellular antibody, validating that its preferential enrichment at presynapse does not originate from nonspecific binding or epitope masking at postsynapse (Fig. 6, *C* and *D*). Other major components of the Kv2 complex, Kv2.2 and AMIGO1, also primarily occupied subsections of synapsin signals but remained distant from PSD-95 (Fig. 6, *E*–*H*). Thus, human Kv2 complex is not uniformly distributed across different synaptic compartments, as it preferentially localizes to the subsynaptic domains of presynaptic terminal but not postsynaptic density.

### Human synaptic terminals recruit smooth ER along with Kv2 proteins

**Figure 7:**
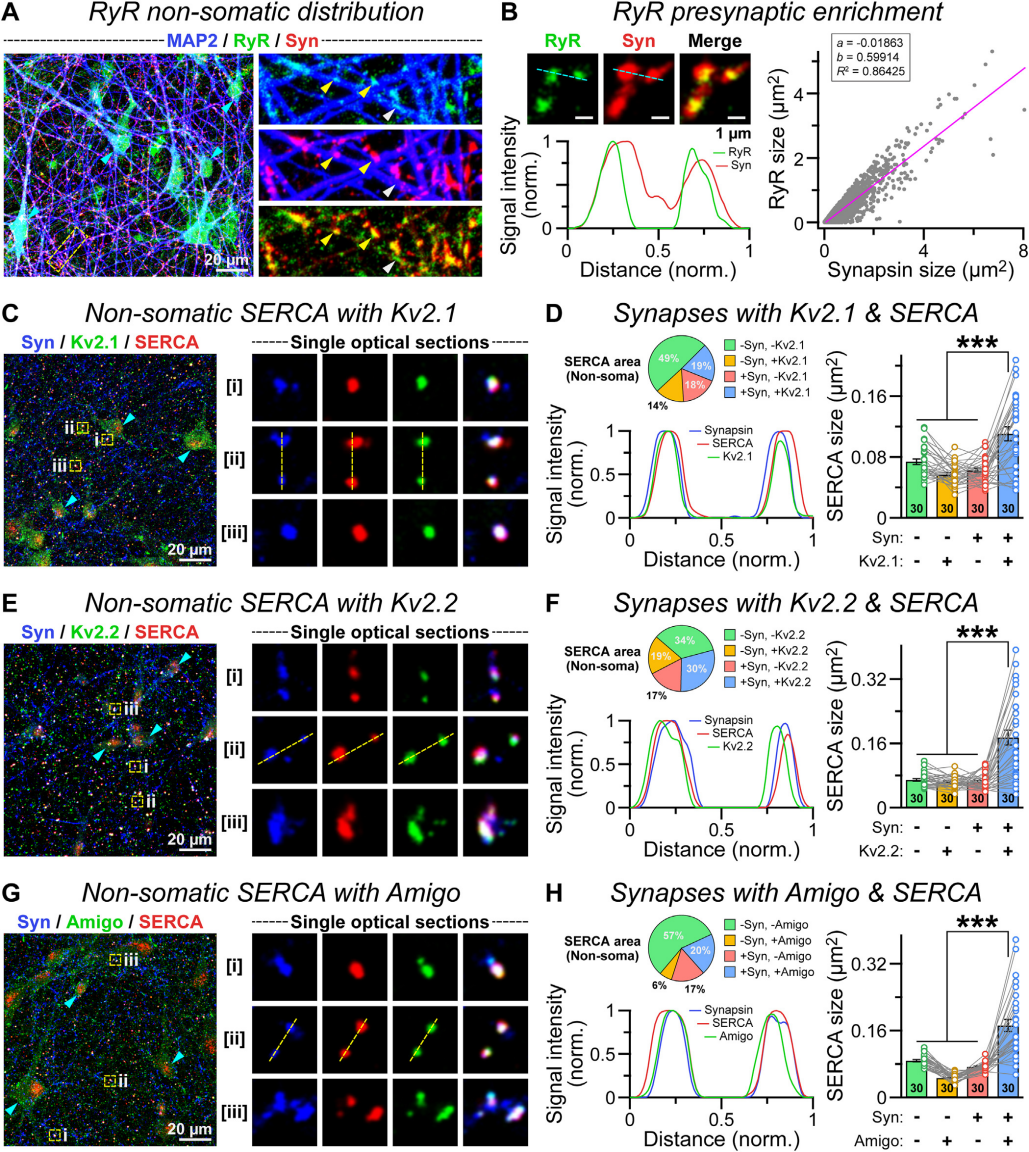
**Association of Kv2 proteins and smooth ER at presynapses.***A*, example of image (*left*) of day 45 human neurons immunostained for MAP2, synapsin, and a smooth ER marker RyR; *cyan arrowheads* = RyRs at cell bodies; the *boxed region* expanded to the *right* depicts nonsomatic RyRs colocalized with synapsin (*yellow arrowheads*) or existed independently (*white arrowhead*). *B*, *left*, a single optical plane (*top*) containing both RyR and synapsin signals or their superimposed views (*merge*); fluorescence intensities (*bottom*) were measured along the *cyan dotted line*. *Right*, relative size of RyR and synapsin clusters, as plotted for individual synapses and fit with a straight line parameter (*purple*). *C*, representative image (*left*) of neuronal cultures immunostained for Kv2.1, synapsin, and a second smooth ER marker SERCA pump; *arrows* point at SERCA signals at soma; *boxed sections* (i, ii, and iii) were zoomed in (*right*) to visualize single synapses within single optical planes containing split channels and merged views. *D*, the relative percentages (*pie chart*, *top left*) of nonsomatic SERCA pumps coinciding with synapsin and/or Kv2.1 area and line intensity profile (*bottom left*) of all three signals calculated across the *yellow dotted lines* in *C*; summary graphs (*right*) provide average size of SERCA clusters, with or without synapsin and Kv2.1. *E* and *F*, same as *C* and *D*, except for the Kv2.2 subunit of human Kv2 complex. *G* and *H*, same as *C* and *D*, except for the AMIGO1 subunit of human Kv2 complex. Summary plots reflect the average (means ± SEM) parameters from each condition, along with total number of field of views (included in bar graphs) analyzed from three to four batches and individual data points (*color-coded open circles*). Statistical evaluations were conducted by one-way ANOVA, with *post hoc* Tukey’s test; ∗∗∗*p* < 0.005. ER, endoplasmic reticulum; MAP2, microtubule-associated protein 2; RyR, ryanodine receptor; SERCA, sarcoendoplasmic reticulum Ca^2+^ transport ATPase.

We asked whether human presynaptic terminals could include smooth ER network, in addition to the ER–PM junction–related Kv2 complex. To assess that, we immunostained day 45 to 50 human neurons for RyRs, that is, an ER-specific Ca^2+^ channel (Fig. 7*A*). We observed that, similar to the Kv2 proteins, RyR is commonly present in cell soma and proximal dendrites, but also concentrates at the peripheral regions of neurons, where they efficiently colocalize with synapsin signals (Fig. 7, *A* and *B*). Notably, the volumes of these nonsomatic RyR clusters increased linearly with that of synapsin-labeled presynaptic terminals, indicating their explicit correlation (Fig. 7*B*).

To further verify the existence of smooth ER structures at presynapses and their association with Kv2 complex, we subsequently labeled the cultures with sarcoendoplasmic reticulum Ca^2+^ transport ATPase (SERCA) antibody paired with both synapsin and individual components of the Kv2 channel. Again, although a large population of nonsomatic SERCA pumps existed outside synapses, representing their general availability at various cellular compartments, a significant level also associated with synapsin in agreement with their synaptic targeting (Fig. 7, *C* and *D*). Especially, the average size of synaptic SERCA signals increased evidently when clustered with Kv2.1 signals (Fig. 7*D*). This phenomenon was broadly consistent with Kv2.2 and AMIGO1 subunits as well (Fig. 7, *E*–*H*), suggesting that human presynaptic terminals can concentrate both smooth ER and ER–PM junction proteins.

### Kv2 traffics to human synapses irrespective of neurogenesis methods

**Figure 8:**
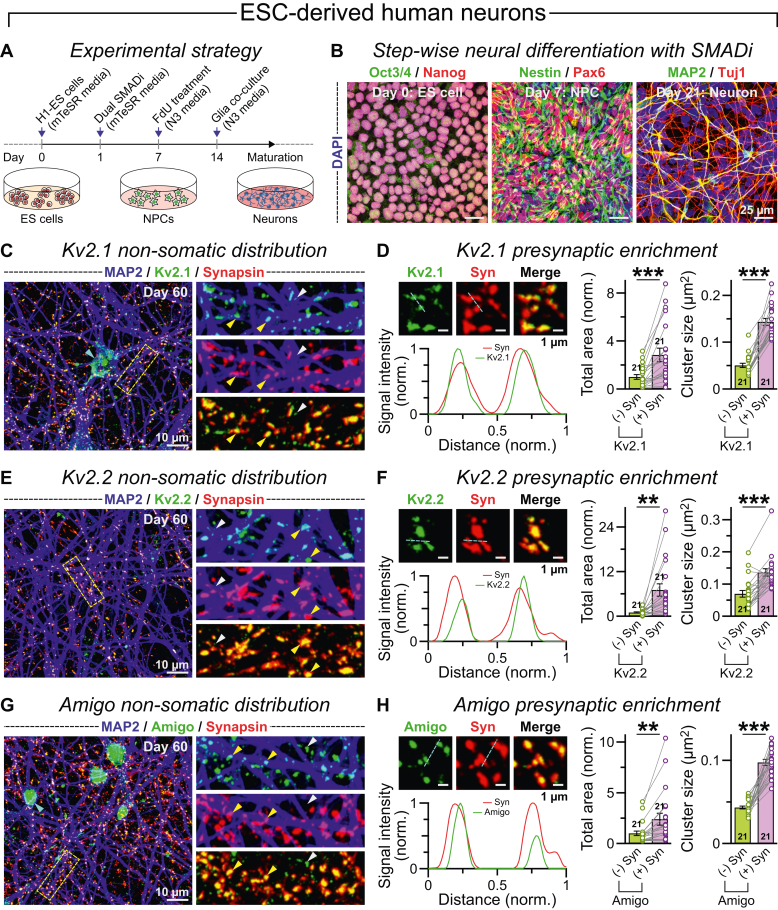
**Synaptic enrichment of Kv2 in ESC-derived human neurons.***A* and *B*, schematic diagram (*A*) and sample images (*B*) of stepwise neurogenesis from human ESCs using small molecules; human ESCs (H1 line; Oct3/4 and Nanog-positive) were treated with bone morphogenic protein and transforming growth factor-β inhibitors to produce neural precursor cells (NPCs; Nestin and Pax6-positive), which were subsequently differentiated into neurons (Tuj1 and MAP2-positive) and cocultured with mouse primary astrocytes for further maturation. *C*, Z-projected example images of ESC-derived human neurons at day 60, immunostained for MAP2, Kv2.1, and synapsin, with *boxed area* further magnified to the *right*; *arrowheads* = Kv2.1 clusters formed on the cell body (*cyan*), or when formed at distant, non-somatic regions, either non-synaptic (*white*) or co-localized with synapsin signals (*yellow*). *D*, single optical sections (*left top*) depicting both Kv2.1 and synapsin puncta, their merged views, as well as normalized signal intensities (*left bottom*) measured along the *dotted line* (*cyan*); *bar graphs* (*right*) indicate total Kv2.1 area or the size of individual Kv2.1 clusters at synapsin negative (−ve) *versus* positive (+ve) regions. *E* and *F*, same as *C* and *D*, except the neurons were immunolabeled with MAP2, Kv2.2, and synapsin antibodies. *G* and *H*, same as *C* and *D*, except the cells were immunolabeled with MAP2, AMIGO1, and synapsin antibodies. All summary graphs reflect means ± SEM and are presented with the total number of field of views analyzed from three to four independent experimental sets. Individual data points are plotted as connected, *color-coded opaque circles*. Statistical significance was assessed by paired, two-tailed, Student’s *t* test (∗∗∗*p* < 0.005; ∗∗*p* < 0.01). ESC, embryonic stem cell; MAP2, microtubule-associated protein 2.

We questioned if the synaptic localization of Kv2 complex was limited to human neurons generated using Ngn2 transcription factor and/or the WTC-11 iPSC line used. To test the reproducibility of our result, we next derived neurons from human embryonic stem cells (ESCs; H1 line) using the dual SMAD inhibition protocol (Fig. 8, *A* and *B*; (63, 64)). We also cocultured the neurons with mouse astrocytes and analyzed them at postdifferentiation day 60. Again, immunostaining for Kv2.1 displayed its robust aggregation at cell body (Fig. 8*C*). However, a sizable fraction of this protein was also located distally and colocalized extensively with synapsin puncta (Fig. 8, *C* and *D*). The total area of these nonsomatic Kv2.1 signals and size of individual clusters also enhanced substantially when accumulated within synapses (Fig. 8*D*). These trends were also phenocopied by both Kv2.2 and AMIGO1 subunits (Fig. 8, *E*–*H*). Taken together, our findings in human neurons supported a consistent enrichment of the Shab family voltage-gated K^+^ channels at presynaptic terminals, which might affect their functional properties.

## Discussion

The Kv2 subfamily ion channels can not only directly modulate neuronal excitability but also facilitate physical contact between the ER and PM, which plays a vital role in Ca^2+^ homeostasis, lipid exchange, membrane protein delivery, and organelle biogenesis (33, 34, 35, 36, 37). Mutations in Kv2 channel proteins often cause severe neurological diseases, including epilepsy, autism, cognitive defect, intellectual disability, and other neurodevelopmental disorders (22, 23, 24, 25, 26), which further highlight its functional impact. Despite this, the subcellular distributions of these Shab family K^+^ channels are relatively unclear and have mostly been characterized in the soma and proximal dendrites of rodent neurons (6, 17, 27, 28, 29, 40, 65). These proteins were shown to generally traffic at astrocytic contact spots, GABAergic postsynapses, but suspected to be excluded from the presynaptic terminals (40, 41, 52), until recently (51).

In this current study, we sought to characterize all major components of endogenous Kv2 channels, Kv2.1, Kv2.2, and their auxiliary subunit AMIGO1, in primarily glutamatergic neurons derived from human stem cells. We provide critical insights into the organization, PTM, and intracellular trafficking of human Kv2 complex and compare our results with existing knowledge in rodent models. Based on our findings, we propose a number of principal conclusions about the molecular properties of the Kv2 complex, especially in a human cellular environment.

We found that several components of the Kv2 channel complex, for example, Kv2.1, Kv2.2, and AMIGO1 subunits, are significantly upregulated during early neurogenesis. They trafficked to the cell surface, interacted with each other, and accumulated at the same cellular regions (Figs. 1 and 4). They also formed fully functional Kv2 channel complexes that operated in a voltage-dependent manner (Fig. 3). The endogenous expression of these proteins was not dependent on human stem cell types or methods employed for neuronal reprogramming, as they were similarly present in both iPSC- and ESC-derived neurons, generated using either transcription factors or small molecules (Figs. 1 and 8). Intriguingly, these Kv2 proteins were particularly enriched in the neuronal population but not cocultured astrocytes (Fig. 4), reflecting their selective presence in specific neural lineages.

Studies in rodents have reported activity-dependent phosphorylation of Kv2.1, which influences both gating properties and VAP binding (31, 55, 56, 57, 58). Although comparable phosphorylation events were predicted for Kv2.2 by generic PKC activation in human embryonic kidney cells and cortical neurons (59), its endogenous PTM status is mostly unclear. Moreover, PTM content of AMIGO1 subunit is also relatively unknown. In human neurons, we noticed a striking difference between the PTM profiles of these different Kv2 components. Although, similar to rodent systems, the human Kv2.1 was evidently phosphorylated but not glycosylated, no major PTM was detected in human Kv2.2; whereas the human AMIGO1 was considerably modified by both phosphorylation and N-linked but not O-linked glycosylation (Fig. 2). Of note, these PTMs in human Kv2.1 and AMIGO1 were not dependent on any neuronal activities but rather reflected a cell-autonomous phenomenon, as they initiated immediately after neurogenesis, that is, at postdifferentiation day 7, much before their functional maturation (60). The contrast in Kv2.2 PTM profile in human *versus* murine models might arise from different developmental stage, activity status, or species context.

Regarding the subcellular localization of Kv2 channel subunits, we noticed that these proteins were highly enriched at cell soma, proximal dendrite, and AIS of human neurons (Fig. 4), similarly to rodent models *in vivo* and *in vitro* (6, 17, 27, 28, 29, 40, 65). However, a sizeable fraction of Kv2 proteins was also localized to more distal parts and efficiently recruited within synapses (Fig. 5). Interestingly, in these cortical layer two-thirds type and purely glutamatergic human neurons, the vast majority of synaptic Kv2.1, Kv2.2, or AMIGO1 clusters were located at presynaptic terminals but remained distant from postsynaptic densities (Fig. 6). This was also fully consistent with two separate Kv2.1 antibodies, against either intracellular or extracellular epitopes. Together, our findings provide strong evidence in support of a recent study that demonstrates robust presynaptic association of both endogenous and overexpressed Kv2 proteins, in rat primary hippocampal cultures (51). Therefore, distribution patterns of Kv2 channels at subsynaptic domains are reproducible and well preserved across multiple species.

The presynaptic terminals of human neurons also contained a substantial amount of smooth ER network, as evidenced by both RyR and SERCA pump recruitment, which directly correlated with presynapse size (Fig. 7). Furthermore, these presynaptic ER clusters exhibited effective colocalization of all major Kv2 subunits (Fig. 7). Using focused ion beam scanning electron microscopy in rodent brain slices, a previous study has reported the presence of ER networks in axon terminals that span entire presynaptic varicosities and often form ER–PM contacts (50). Although several proteins can establish ER–PM junctions in both excitable and nonexcitable cell types, for neurons, these specialized structures could be primarily composed of the Kv2 family proteins (28, 29, 66, 67, 68). The obvious enrichment of both smooth ER markers and ER–PM junction–associated Kv2 channels at human presynapses, as well as their striking colocalization with enlarged volumes, might suggest the possible existence of presynaptic ER–PM junctions also in human neurons, again reflecting a conserved phenomenon.

Our model system provides a robust and consistent platform to study the Kv2 channel properties in human cellular context, because human neurons generated from stem cells can be induced to acquire homogeneous identities, especially when reprogrammed using lineage-specific transcription factors. In the future, this model can be further utilized to examine basic cell biological questions about Kv2 complex organization, their conducting or nonconducting roles including relative contribution in forming ER–PM contact sites, signaling pathways that potentially modulate their PTM status and alter channel functionalities, as well as molecular mechanisms that guide their trafficking and recruitment at specific subcellular compartments including the presynaptic terminals.

## Experimental procedures

### Cell lines

ESC (H1 line; WiCell) and iPSC (WTC-11 line; gifted by Dr Michael E. Ward, National Institute of Neurological Disorders and Stroke) culture procedures were authorized by the Institutional Biosafety Committee (protocol #1−059B), Colorado State University. Dissection of mouse brain (C57BL/6 strain) for astrocytes was approved by the Institutional Animal Care and Use Committee (protocol #5237).

### Stem cell culture

ESCs or iPSCs were plated on 6-well dishes precoated with Matrigel (BD Biosciences) and cultured under feeder-free conditions in mTeSR1 or mTeSR Plus media (StemCell Technologies), which was exchanged every day. At 60 to 70% confluency, they were dissociated with PBS + 0.5 mM EDTA and passaged at a 1:6 dilution until ready for differentiation. During passages, the media was also supplemented with ROCK-inhibitor Y-27632 (2.5 μM; MedChem Express) but excluded thereafter.

### Generation of neurons from iPSCs

iPSCs were plated at 1:12 dilution in N3 media (composition: Dulbecco's modified Eagle's medium/F12 [Thermo Fisher] + N2 [Thermo Fisher] + B27 [Thermo Fisher] + insulin [20 μg/ml, Sigma–Aldrich] + penicillin–streptomycin mix [1%, Thermo Fisher]). The next day, doxycycline (2 μg/ml, Sigma–Aldrich) was added to the media to induce Ngn2 expression and trigger neurogenesis. After 5 to 7 days, cells were dissociated with EDTA, mixed with passage 1 to 2 mouse primary glia, and replated on Matrigel-coated glass coverslips placed inside 24-well dishes. During the first 24 h, N3 media contained 10% fetal bovine serum (Altas Biologicals), which was consecutively diluted to a final concentration of 2.5% by half replacements over the next 3 to 4 days, when equal volumes of media were added without any serum. 5-Fluorodeoxyuridine (FdU, 10 μM) was included in the media to inhibit glial proliferation after reaching ≈70 to 80% confluency. After 15 days of culture, the media were gradually switched to Neurobasal Plus (Thermo Fisher) media, containing B27 (Thermo Fisher), 2.5% fetal bovine serum, 10 μM FdU, and 1% penicillin–streptomycin, by half-exchanges every 3 to 5 days.

### Generation of neurons from ESCs

Differentiation of ESCs into neural precursor cells was achieved by dual SMAD inhibition (63), with minor modifications as described previously (60, 64). In brief, human ESCs cultured in mTeSR1 media were treated with LDN193189 (100 nm; StemCell Technologies) plus SB431542 (10 μm; StemCell Technologies) for 6 to 7 days to induce neural precursor cell identity. They were subsequently passaged and expanded by 1:6 splits, then treated with FdU to prevent further proliferation, and gradually transitioned into N3 media. During this period, the NSCs spontaneously differentiated into Tuj1-positive neurons. They were finally dissociated with Accutase (Innovative Cell Technologies) and replated with mouse glia on Matrigel-coated coverslips at a 1:50 ratio. Starting around day 30 of culture, the media was slowly switched into Neurobasal Plus media.

### Immunoblotting

Cells were lifted and dissociated with PBS + EDTA and then pelleted by spinning at ∼800*g* for 5 to 10 min. The pellets were lysed with radioimmunoprecipitation assay (RIPA) buffer (Thermo Fisher) supplemented with Halt protease inhibitor cocktail (Thermo Fisher) for 1 h. The lysates were then centrifuged at ∼800*g* for 15 min, and supernatants containing the protein extracts were collected, aliquoted, and stored at −80 °C for future use.

Lysates were diluted if needed to adjust concentrations before adding 4x Laemmli Sample Buffer (Bio-Rad) supplemented with SDS and β-mercaptoethanol. The proteins were run on 4% stacking and 7.5% resolving SDS-PAGE for ∼2.5 h and transferred to nitrocellulose membranes for 1 h. Membranes were blocked overnight while rocking at 4 °C, in Tris-buffered saline (TBS) containing 5% nonfat milk and 5% bovine serum albumin, supplemented with 0.1% Tween-20 detergent (TBST). Blots were incubated with primary antibodies dissolved in blocking buffer for 2 to 3 h at room temperature, washed four times with TBST, incubated with blocking buffer containing secondary antibodies (1:3000 dilution; DyLight 680/800; Invitrogen) for another 1 to 2 h at room temperature, and then washed again four times with TBST. Membranes were imaged immediately using a LI-COR Odyssey CLx system; signal contrast and brightness were uniformly adjusted by the Image Studio software (version 5.2; LI-COR). Protein contents and band positions were estimated by plotting intensity profiles for each lane and calculating the peak areas underneath.

### Surface biotinylation

Cell surface proteins consisting of primary amines were tagged with biotin using the Pierce biotinylation and isolation kit (Thermo Fisher) following the manufacturer’s protocol. Briefly, live neuronal cultures were incubated with PBS containing sulfo-NHS-SS-biotin for 10 min at room temperature and then washed with TBS to quench the reaction. Cells were mechanically lifted, pelleted, and lysed. To extract biotin-labeled proteins, the total lysate was incubated with NeutrAvidin agarose beads, and the initial flow-through was saved as unbound intracellular fraction. The beads were then washed thoroughly, and biotinylated surface proteins were eluted out.

### Immunoprecipitation

The whole-cell lysates in RIPA buffer + protease inhibitor cocktail were mixed with primary AMIGO1 antibody or an immunoglobulin G control (≈1 μg each) while rotating overnight at 4 °C. This mixture was subsequently incubated for another 4 h with Dynabeads Protein G (Invitrogen) at 4 ^°^C. These beads were then washed three times with RIPA buffer, and the proteins plus antibodies were dissociated from the beads with SDS sample loading buffer and 10 mM DTT. The eluates were collected, run on SDS-PAGE, and probed for Kv2.1 and AMIGO1.

### **λ**-Phosphatase treatment

The cell lysates were combined with 10x protein metallophosphatase buffer, 10 mM MnCl_2_, and λ protein phosphatase in ratios according to the manufacturer’s direction (New England Biolabs; catalog no.: P0753). In control conditions, an equal volume of water was added instead of λ protein phosphatase. Samples were mixed thoroughly, incubated for 3 h at 30 °C while shaking, and subsequently run on Western blot.

### Glycosidase treatment

Glycosidase treatments were performed similarly to our previous report (69). In brief, whole-cell extracts were combined with 10x GlycoBuffer2, PNGase F (New England Biolabs; P0709), O-glycosidase (New England Biolabs; P0733), or both enzymes combined, in ratios according to the manufacturer’s protocol. In control conditions, an equal amount of water was added instead of glycosidase. The samples were mixed adequately, incubated overnight at 37 °C on a tube rotator, and then run on Western blot the next day.

### Immunocytochemistry

The neuronal cultures were rinsed once with PBS only and then immediately fixed by 4% paraformaldehyde for 30 to 45 min at room temperature. They were washed three more times with PBS and blocked by 10% HyClone cosmic calf serum + 0.1% Triton X-100 detergent dissolved in PBS for 1 h while rocking at 37 °C. For immunolabeling the extracellular epitope of Kv2.1 at the cell surface, the blocking buffer was prepared without the membrane permeabilization agent Triton X-100. Next, primary antibodies were mixed in the blocking buffer at specific concentrations, added to the samples, and incubated for 2 to 3 h while rocking at 37 °C. After the primary antibody incubation, cultures were washed four times and then exposed to blocking buffer containing secondary antibodies conjugated with Alexa-Fluor dye (405/488/546/647; Invitrogen) for 1 to 2 h at 37 °C. Following this, coverslips were washed four more times with the blocking buffer and once with PBS only. For nuclear staining, cells were further incubated with DAPI diluted in blocking buffer for 10 min at 37 °C and then washed twice with PBS. The coverslips were mounted upside down on glass slides using Fluoromount-G (Southern Biotech) and left to solidify overnight at room temperature, prior to confocal imaging.

### Confocal microscopy

All images were acquired using an inverted STELLARIS 5 (Leica Microsystems) laser scanning microscope with 405/488/561/638 nm wavelengths containing Power HyD detectors. Samples were imaged through a series of 0.5 μm thick optical z-sections under 40x (1.3 numerical aperture) and 63x (1.4 numerical aperture) oil immersion, plan-apochromatic objectives. The images were processed through Leica Application Suite version X (LasX) and analyzed using ImageJ (FIJI, National Institutes of Health) software. Images from regions of interest were presented as a single optical section or superimposed as maximum-intensity z-projections (10–20 slices). The colocalization parameters between two fluorescent signals were assessed by first thresholding individual channels appropriately to eliminate any background noise and then measuring pixel-based percentage of coincidence between their projected areas.

### List of antibodies

Antibodies selected for Western blot and/or immunostaining assays included appropriate combinations of mouse anti-Kv2.1 (intracellular epitope, 1:250 dilution; Antibodies, Inc, catalog no.: NeuroMab K89/34), mouse anti-Kv2.1 (extracellular epitope, 1:250 dilution; DSHB, catalog no.: NeuroMab K39/25), mouse anti-Kv2.2 (1:250 dilution; DSHB, catalog no.: NeuroMab K37/89), mouse anti-AMIGO1 (1:250 dilution; Antibodies, Inc, catalog no.: NeuroMab L86A/37), mouse anti-GAPDH (1:5000 dilution; ProteinTech, catalog no.: 60004), chicken anti-MAP2 (1:500–1000 dilution; Abcam, catalog nos.: ab92434 and ab5392), rabbit anti-Ankyrin-G (1:500–1000 dilution; Thermo Fisher, catalog no.: PA5-143596), rabbit anti-SERCA2 (1:500–1000 dilution; Abcam, catalog no.: AB3625), mouse anti-RYR1/RYR2 (1:500–1000 dilution; DSHB, catalog no.: 34C), rabbit anti-Synapsin1/2 (1:1000 dilution; Synaptic Systems, catalog no.: 106002), guinea pig anti-Synapsin1/2 (1:1000 dilution; Synaptic Systems, catalog no.: 106004), mouse anti-Tuj1 or β-tubulin III (1:500 dilution; BioLegend, catalog no.: 801202), mouse anti-Oct3/4 (1:500–1000 dilution; Santa Cruz Biotechnology, catalog no.: sc-5279), mouse anti-Nestin (1:300 dilution; R&D Systems, catalog no.: MAB1259), rabbit anti-Nanog (1:250 dilution; Abcam, catalog no.: ab21624), rabbit anti-Pax6 (1:300 dilution; Covance, catalog no.: PRB-278P), and a fluorophore-labeled anti-PSD-95 (1:500 dilution; Synaptic Systems, catalog no.: N3702-AF647-L). DAPI stain (1:50,000 dilution; Thermo Fisher, catalog no.: D1306) was used for nuclear labeling.

### Electrophysiology

Whole-cell patch-clamp recordings were performed similarly to those described previously (70). In brief, the reprogrammed human neurons were patched in voltage-clamp mode using internal solution consisting of (in millimolar) ≈ 120 KCl, 5 NaCl, 10 EGTA, 1 MgCl_2_, 10 Hepes, 3 Mg–ATP, and 0.3 Na–GTP; 310 mOsm, and pH adjusted at 7.3 to 7.4 using KOH. The extracellular solutions contained the following (in millimolar) ≈ 140 NaCl, 5 KCl, 2 CaCl_2_, 2 MgCl_2_, 10 glucose, and 10 Hepes; 300 mOsm, and pH adjusted at 7.3 to 7.4 with NaOH. The electrophysiological recordings were conducted using an integrated patch-clamp amplifier (Sutter Instrument) with a customized Igor Pro (WaveMetrics) data acquisition system. Recordings were conducted at a holding potential (V_hold_) = −70 mV and then incremented stepwise by 5 mV for 500 ms, up to V_hold_ = +50 mV.

The external solution contained CNQX (50 μM, α-amino-3-hydroxy-5-methyl-4-isoxazolepropionic acid receptor blocker; Tocris Bioscience), CPP (50 μM, *N*-methyl-d-aspartate receptor blocker; Tocris Bioscience), and picrotoxin (100 μM, GABA_A_/glycine receptor blocker; Tocris Bioscience) to inhibit all spontaneous synaptic currents. After achieving a stable patch, whole-cell currents for voltage-gated K^+^ and Na^+^ channels were recorded before and after bath applications of Guangxitoxin−1E (100-200 nM; catalog no.: STG-200, Alomone Labs) or Stromatoxin-1 (100–200 nM; catalog no.: STS-350, Alomone Labs).

### Data presentation

For all figure panels, the average values (*bar graphs*, *symbols* [*circles* or *squares*], or *pie charts*) reflect means ± SEM (*i.e.*, standard deviations of a given parameter divided by square root of sample numbers) and are presented with number of replicates, for example, cultures examined (immunoblots) or field of views imaged (immunostaining) from independent experimental batches. Individual data points are included as *color-matched symbols*, connected with *lines* for paired comparison. The type and strength of statistical evaluations between groups are mentioned in corresponding figure legends. This included paired (batchwise assessments) or unpaired (multiple measurements from each batch), two-tailed, Student’s *t* test, and one or two-way ANOVA with replications; ∗∗∗*p* < 0.005; ∗∗*p* < 0.01; ∗*p* < 0.05; ns = nonsignificant, *p* > 0.05.

### Data availability

All individual data points are provided in the main figures along with their corresponding average quantifications. The raw data are available upon request to S.C. (soham.chanda@colostate.edu). Requests for experimental reagents should be addressed to S.C. (soham.chanda@colostate.edu).

## Conflict of interest

The authors declare that they have no conflicts of interest with the contents of this article.
